# m6A modification in RNA: biogenesis, functions and roles in gliomas

**DOI:** 10.1186/s13046-020-01706-8

**Published:** 2020-09-17

**Authors:** Yuhao Zhang, Xiuchao Geng, Qiang Li, Jianglong Xu, Yanli Tan, Menglin Xiao, Jia Song, Fulin Liu, Chuan Fang, Hong Wang

**Affiliations:** 1 Department of Neurosurgery, Affiliated Hospital of Hebei University, 071000 Baoding, China; 2 Faculty of Integrated Traditional Chinese and Western Medicine, Hebei University of Chinese Medicine, 050091 Shijiazhuang, China; 3 Faculty of Acupuncture-Moxibustion and Tuina, Hebei University of Chinese Medicine, 050200 Shijiazhuang, China; 4 Department of Pathology, Affiliated Hospital of Hebei University, 071000 Baoding, China; 5 School of Basic Medicine, Hebei University, 071000 Baoding, China; 6 Office of Academic Research, Affiliated Hospital of Hebei University, 071000 Baoding, China; 7 Hebei Key Laboratory of Chinese Medicine Research on Cardio-Cerebrovascular Disease, Hebei University of Chinese Medicine, 050091 Shijiazhuang, China

**Keywords:** m6A modification, RNA, Central nervous system, Glioma, Glioblastoma, GBM, Tumourigenesis

## Abstract

The chemical modification of RNA is a newly discovered epigenetic regulation mechanism in cells and plays a crucial role in a variety of biological processes. N6-methyladenine (m6A) mRNA modification is the most abundant form of posttranscriptional RNA modification in eukaryotes. Through the development of m6A RNA sequencing, the relevant molecular mechanism of m6A modification has gradually been revealed. It has been found that the effect of m6A modification on RNA metabolism involves processing, nuclear export, translation and even decay. As the most common malignant tumour of the central nervous system, gliomas (especially glioblastoma) have a very poor prognosis, and treatment efficacy is not ideal even with the application of high-intensity treatment measures of surgery combined with chemoradiotherapy. Exploring the origin and development mechanisms of tumour cells from the perspective of tumour biogenesis has always been a hotspot in the field of glioma research. Emerging evidence suggests that m6A modification can play a key role in gliomas through a variety of mechanisms, providing more possibilities for early diagnosis and targeted therapy of gliomas. The aim of the present review is to focus on the research progress regarding the association between m6A modification and gliomas. And to provide a theoretical basis according to the currently available literature for further exploring this association. This review may provide new insights for the molecular mechanism, early diagnosis, histologic grading, targeted therapy and prognostic evaluation of gliomas.

## Background

Gliomas are the most common malignancy in the central nervous system. Glioblastoma (GBM) has the highest malignancy rate and account for 50% of all brain tumours. The average survival time of patients with GBM is only 14.6 months [1]. GBM originate from poorly differentiated glial cells and have the characteristics of nuclear atypia, cellular polymorphism, and a high degree of mitotic activity. Given the aggressiveness of GBM, surgical resection to resolve all intracranial lesions in clinical practice is challenging. Therefore, most patients receive radiotherapy and temozolomide (TMZ) combined with chemotherapy after surgery. Although the treatment intensity is very high, the outcomes are still not satisfactory [2–5]. Therefore, there is an urgent need for new treatment strategies or regimens.

To improve the efficacy of GBM treatment, it is necessary to understand the occurrence and development of GBM and determine the molecular biological characteristics of GBM. In recent years, with the in-depth study of the epigenetics, metabolism, and immunology of GBM, our knowledge of GBM has greatly expanded, which provides new clues for the treatment of GBM. Recent studies have found that non-coding RNAs and post transcriptional modification of RNAs have become the active fields of cancer research. Among them, N6-methyladenine (m6A) RNA modification is the important research hotspot [6, 7]. More than 60% of all RNA modifications are methylated modifications, and m6A is the most abundant chemical modification in eukaryotic messenger RNA (mRNA). As a representative of relevant studies, epigenetics found that m6A RNA modification plays important roles in the regulation of cell fate, proliferation, and metabolism and the biogenesis of tumours [6–9]. It also opens a new way of thinking for biomedical scientists, whether RNA modification will be another important factor to regulate the biological development of neoplastic diseases.

In this review, we provide a comprehensive introduction of the latest research progress on m6A modification and elucidate the origin of m6A modification, its regulation, biological functions and its correlation with the central nervous system and gliomas. And to discuss the prospect of the possible research directions. Aiming at providing a theoretical basis according to the currently available literature for further exploring the association between m6A modification and gliomas.

## The molecular mechanisms of m6A modification

### Origin of m6A modification

With the advent of high-throughput sequencing technology, scientists have identified nearly 170 types of RNA modification, also known as “epitranscriptomics” [10]. Inserting information beyond the information carried by their base sequences to gene transcripts, altering the charge of RNA bases and their matching properties, differential folding of RNAs, and the formation of a regulatory protein-RNA interaction recognition element are classical transcriptome modification processes. Therefore, these modifications are involved in the whole process of RNA metabolism, regulating the fine expression patterns of genes and, ultimately, affecting the biological behaviour of cells [11, 12].

When the m6A modification was first discovered in mRNA, only a few modified sites were mapped in viral and cellular RNAs, but not in the human coding region or noncoding region [13]. In 2012, Dominissini and colleagues developed m6A sequencing (m6A-seq) technology. They presented the first modification landscape in a transcriptome-wide manner using m6A-seq, proved the evolutionary conservation of m6A sites and identified dynamically modulated sites in responses to stimuli in cells. Gene sequence analysis showed that in the consensus sequence RRACH (in which R represents A or G and H represents A, C or U), m6A modification usually occurs [14].

### Approaches of detecting of m6A modification

The total amount of m6A in RNA can be probed by several approaches, including two-dimensional thin layer chromatography, m6A dot-blot and high-performance liquid chromatography-tandem mass spectrometry (HPLC-MS/MS) [15, 16].

The transcriptome-wide distribution of m6A was unclear before 2012, until methylated RNA immunoprecipitation followed by high-throughput sequencing (MeRIP-seq or m6A-seq) was developed [17, 18]. In this method, mRNA was fragmented into 100-nt-long oligonucleotides and immunoprecipitated with a specific antibody against m6A. Then immunoprecipitated RNAs were subjected to high-throughput sequencing. Other approaches for higher resolution, such as photo-crosslinking-assisted m6A-sequencing (PA-m6A-Seq) and site-specific cleavage and radioactive-labelling followed by ligation-assisted extraction and thin-layer chromatography (SCARLET) [19, 20]. Currently, a novel method called m6A individual nucleotide resolution crosslinking immunoprecipitation (miCLIP) has marked a major step forward in the field, which could detect m6A at precise position [21]. Furthermore, with the development of CRISPR-based genome engineering, it is currently possible to directly detect the effect of altering any m6A modification site in many organisms. As a supplementary approach, it would be valuable for discovering the functions of m6A methylation [22, 23].

Current studies have shown that in addition to mRNA, microRNA (miRNA), long noncoding RNAs (lncRNA), circular RNA (circRNA), ribosomal RNA (rRNA), transfer RNA (tRNA) and small nucleolar RNAs (snoRNA) all have m6A modifications and that their regulation involves almost all categories of protein-coding genes and noncoding genes [24, 25].

### Regulation of m6A modification

**Fig. 1:**
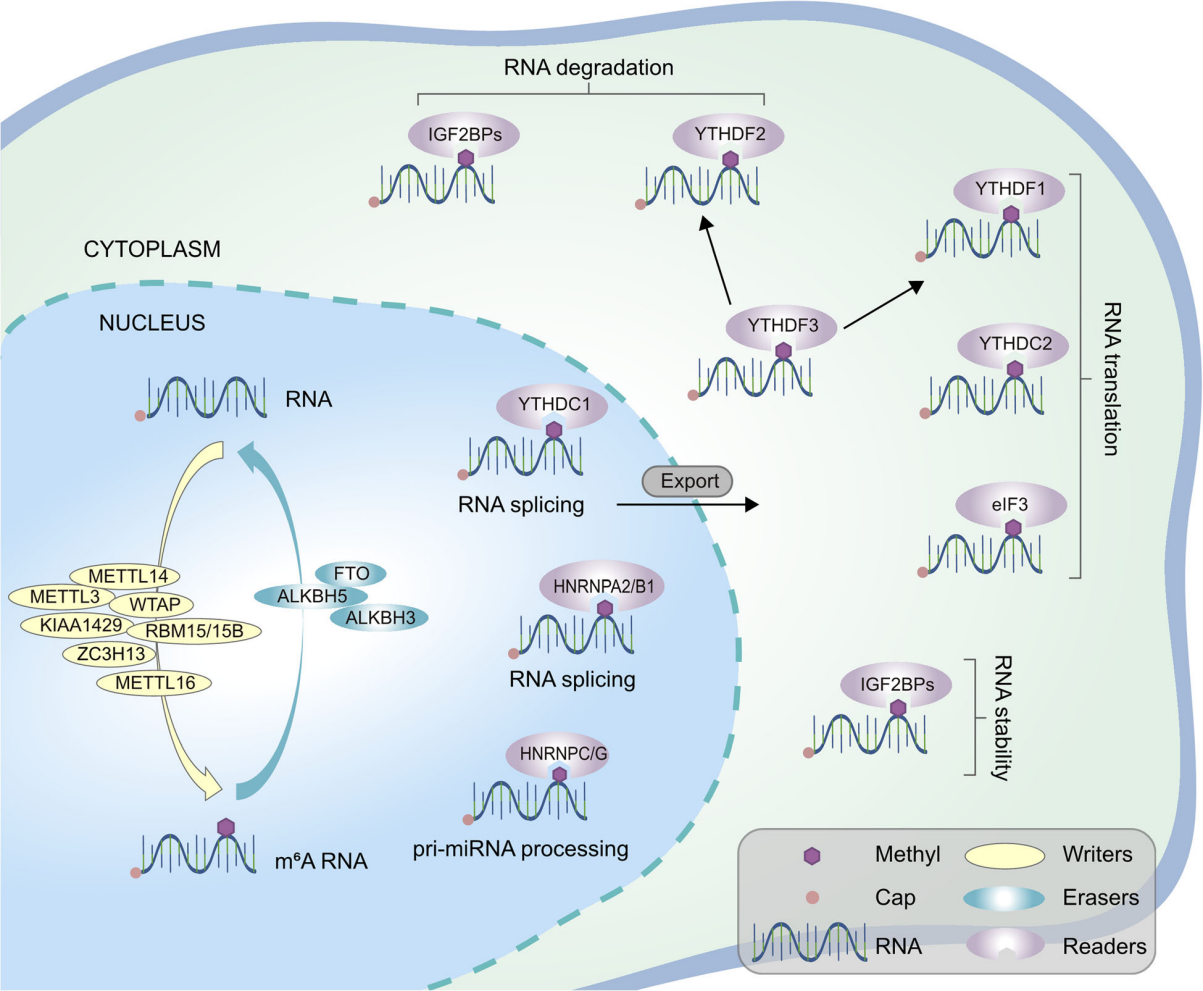
The molecular mechanism of RNA m6A modification. m6A is installed by “Writers” (METTL3/14/16, WTAP, KIAA1429, RBM15/15B and ZC3H13), removed by “Erasers” (FTO, ALKBH3 and ALKBH5), and recognized by “Readers” (YTHDC1/2, YTHDF1/2/3, IGF2BPs, HNRNPA2/B1 and eIF3)

m6A modification mainly occurs in adenines in the RRACH sequence, and its functions are implemented by RNA methyltransferases (writers), RNA demethylase (erasers) and m6A binding protein (readers). We summarized the types of proteins involved in m6A modification (Fig. 1). In addition, we offered some explanations for some of these important biological functions.

#### m6A “writer”

Methyltransferase like 3 (METTL3) is a key protein with molecular weight of 70 kDa and was the first protein identified as an “m6A writer” [26–28]. Gel filtration chromatography shows that METTL3 and METTL14 can form a stable and asymmetric METTL3-14 complex with a stoichiometric ratio of 1:1; then, this complex combines with Wilms’ tumour 1-associating protein (WTAP) to play a methylation function [27, 29]. In addition, METTL3 can play a central catalytic role towards methyl groups, allowing these groups to partially transfer from the S-adenosylmethionine (SAM) moiety to the receptor adenine. At the same time, METTL14 plays an important role in promoting substrate binding [30]. WTAP interacts with the METTL3-14 complex to affect m6A methyltransferase activity and localization in nuclear speckles [26, 27].

#### m6A “eraser”

After fat mass and obesity-associated gene (FTO) knockdown, the m6A level in mRNA increases [31]. Meyer C et al. noted that FTO has a high affinity for m6A modifications; because m6A is a reversible RNA modification, FTO can affect the fate of cellular mRNA. In this process, FTO preferentially demethylates m6A and reduces the stability of the m6A-related mRNA [32]. AlkB homologue 5, RNA demethylase (ALKBH5) resides in the nucleus, and the m6A level in mRNA is significantly reduced in cells that overexpress ALKBH5. ALKBH5’s depletion affects mRNA export and assembly processes [33].

#### m6A “reader”

YTH N6-methyladenosine RNA binding protein 1 (YTHDF1) promotes the translation of m6A-modified mRNA, YTHDF2 accelerates RNA decay, and YTHDF1/2 and YTHDF3 synergistically promote RNA metabolism in the cytoplasm [34–36]. In the localization of nuclear speckles, under the action of YTH domain containing 1 (YTHDC1), serine-rich splicing factor 3 (SRSF3) expression is promoted, and SRSF10 expression is inhibited, thereby regulating the splicing of mRNA [37]. In addition, YTHDC2 preferentially binds to m6A-containing transcripts, thereby reducing mRNA abundance and improving translation efficiency through interactions with translation initiation and decay mechanisms [38].

## Biological functions of m6A modification

### m6A modification involved in RNA metabolism

**Fig. 2:**
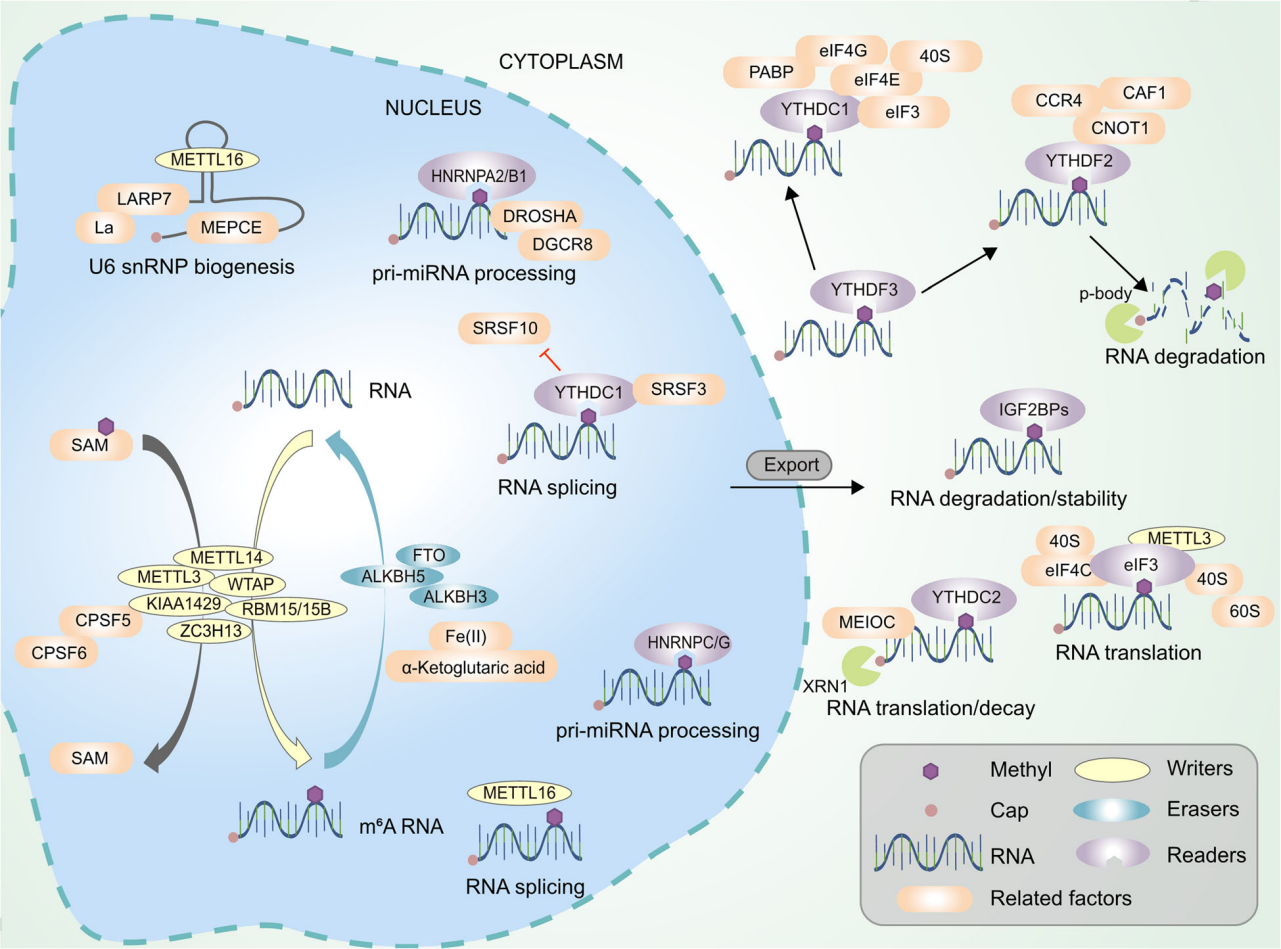
The functions of RNA m6A modification related proteins. “Writers”, “Erasers” and “Readers” relay on some important factors install, remove and recognize m6A modification and participate in a variety of steps in RNA metabolism, including splicing, export, translation, degradation, decay and so on

m6A modification is involved in the regulation of almost all processes of RNA metabolism. For mRNA, m6A is involved in the regulation of the processing and expression of pre-mRNA (precursor RNA) in the nucleus and the translation and decay of mature mRNA in the cytoplasm (Fig. 2) [39]. The regulation of the alternative splicing of precursor mRNA [35] is achieved through the activation of the SRSF3 pathway by the binding of m6A to YTHDC1 in the nucleus, which mainly involves the exons at the ends of mRNA. This process is also the regulatory mechanism for the diversity of adenosine polymer [40, 41]. METTL3, ALKBH5, and YTHDC1 also play important roles in the regulation of mRNA nuclear export [33, 37]. In addition, the diverse mechanism of translation regulation is a major function of m6A in the cytoplasm. m6A can not only regulate the translation efficiency of mRNA through the YTHDF1-eIF3 pathway [34] but can also mediate related processes through insulin-like growth factor-II mRNA binding protein (IGF2BP) [42]. During cellular stress responses, signalling pathways regulating 5’-end cap structure independent translation is also dependent on m6A [43]. Second, m6A also plays an important role in maintaining mRNA stability [42], of which the mechanism mainly involves the recruitment of mRNA into the processing bodies (P body) through YTHDF2, followed by biological degradation [44]. Liu et al. also found that m6A can fully participate in the entire RNA metabolism process through the regulation of the RNA secondary structure; furthermore, this function of m6A modification is also closely related to the biogenesis and development of neoplastic diseases [45, 46].

### m6A modification regulates the directed differentiation of haematopoietic stem cells

METTL3-mediated m6A modification is involved in the regulation of multiple biological functions in eukaryotic organisms. In recent years, relevant studies have found that it is closely related to biological rhythm, stem cell self-renewal, maternal-to-zygotic transition, DNA damage response, neurological function regulation and sex determination in *Drosophila* and early mouse embryonic development [47–56].

Zhang et al. completed the m6A modification profile for zebrafish embryogenesis and found that in the process of EHT, as a key factor, neurogenic locus Notch homologue protein 1 (Notch1a) under m6A-specific modification initiates the binding of YTHDF2 and mRNA decay, resulting in the inhibition of the Notch pathway, thereby allowing the programmed occurrence of aforementioned biological behaviours and ultimately endothelial cell transition into haematopoietic stem cells (HSPCs) [57]. In addition, after METTL3-specific knockout in mouse embryos, similar functional phenotypes were observed [58]. In general, m6A modification plays an important regulatory role in the process of vertebrate HSPC differentiation and even in the blood development process.

### m6A modification and the maintenance of embryonic stem cell (ESC) pluripotency

Epigenetic and epitranscriptomic networks play important roles in somatic cell reprogramming and in the maintenance of ESC pluripotency. Recent studies have shown that zinc finger protein 217 (ZFP217) activates the transcription of key pluripotency genes and regulates m6A deposition on transcripts. Zfp217 depletion generally enhances the m6A modification of Nanog, sex determining region Y box 2 (Sox2), Kruppel-like factor 4 (Klf4) and c-Myc mRNAs, thereby accelerating their decay and directly destroying ESC self-renewal and somatic cell reprogramming [51, 52]. This finding reveals the close relationship between m6A and somatic cell reprogramming and the maintenance of ESC pluripotency.

### m6A modification and the regulation of spermatogenesis

Geula et al. found that METTL3-specific knockout in mice leads to early embryonic lethality [52]. Using the CRISPR/Cas9 and Cre-loxP systems, Xu et al. constructed a mouse model based on homologous recombination technology, specifically the knockdown of METTL3 in germ cells (Vasa-Cre). Haematoxylin-eosin (HE) staining and immunofluorescence staining showed that the differentiation and meiosis of spermatogonial stem cells in male mice were significantly inhibited, leading to infertility. In addition, the researchers also found that after METTL3 knockout, not only was the m6A RNA modification level reduced but also altered RNA alternative splicing and expression profiles were observed in the genes functioning in the maintenance of spermatogonial stem cell differentiation and cell meiosis, seriously affecting the biological formation of gametes [59].

In addition, using Vasa-Cre technology, Lin et al. specifically knocked out METTL14 in the germ cells of mice, significant disruption of the translation function of transcription products of spermatogonial stem cell proliferation and differentiation was also observed, eventually leading to the depletion of spermatogonial stem cells. Stra8-GFP Cre technology was used for the simultaneous knockout of METTL3 and METTL14, the coding and translation of haploid-specific genes was incorrect, and sperm production was inhibited [60]. On the other hand, animal experiments showed that model mice presented testicular shrinkage, spermatogenesis abnormalities, and poor sperm motility after ALKBH5 overexpression [33]. After YTHDC2 knockout, apoptosis occurred in spermatogonial cells at the early stage of meiosis, resulting in testicular shrinkage and spermatogenesis disorder [36, 61]. In summary, maintaining m6A modification homeostasis is critical for the regulation and protection of normal sperm development.

### m6A modification and the regulation of brain development

Animal experiments showed that the abundance of m6A was significantly higher in the cerebellum than in the cerebral cortex of mice, suggesting that m6A may play a critical role in this anatomical region [62]. Wang et al. studied the role of m6A modification in the development of the central nervous system. Scientists used the Cre-loxP system to silent the METTL3 gene in the brains of mice. The study found that the model mice had severe movement disorders during the lactation period; some mice even died. Morphological observation of dead mice revealed severe dysplasia in both the cerebral cortex and cerebellar area. Mice in which the METTL3 gene was silenced showed severe cerebellar hypoplasia, which might be associated with the rapid apoptosis of cerebellar granule cells (CGCs) in the external granular layer (EGL) of new-born mice [63].

Studies have shown that the dynamic process of m6A modification occurs throughout the entire process of cerebellum development after a mouse is born. In the case of hypobaric hypoxia, the presence of ALKBH5 gene deletion directly lead to altered m6A levels during cerebellar development and accelerated RNA nuclear export, seriously affecting the developmental process of the cerebellum [64]. In addition, after METTL14-specific knockout, the development of the mouse cerebral cortex was also severely impaired [65]. Li et al. found that the deletion of the YTHDF2 gene in mice not only improved the level of m6A modification but also inhibited the normal RNA decay of the genes and proteins involved in neural stem cell differentiation and neuron dendrite formation; therefore, neural stem cells were unable to divide, the number of neural precursor cells was seriously insufficient, and the differentiation of mature neurons was severely affected, thereby affecting the development of brain tissues [66]. The above studies revealed that methylation and demethylase-mediated posttranscriptional m6A modification play key roles in the development of the mammalian central nervous system, especially cerebellar development.

### m6A modification involvement in the development of nerve cells and neural regulation in adults

After axon injury, the level of m6A modification in nerve cells increases, and the protein translation efficiency of many related genes, including axonal regeneration-related genes, significantly improves [63]. Studies have shown that the expression of the important “eraser” of m6A, FTO, is highest in the cerebral cortex and is highly expressed in mature neural stem cells and neurons [67] and that FTO deletion seriously damages the neurogenesis and cognitive function of adults [68]. Furthermore, FTO has a highly dynamic expression profile, and researchers suspect that FTO may play a key role in the central nervous system by regulating the expression of m6A-modified nerve-related genes. In addition, RNA methylation also plays a role in the regulation of synaptogenesis in some areas to participate in learning and memory [69–72]. Generally, the dynamic equilibrium of RNA chemical modification is the most important feature in nervous system function. m6A RNA modification also plays an important role in the formation, differentiation, and self-renewal process of mature neurons [67].

### Other important functions of m6A modification

Studies have shown that if the m6A modification level of mRNAs of suppressor of cytokine signalling (SOCS) family genes declines, mRNA decay of naïve T cells can be delayed, and the protein expression levels of SOCS1/3 and cytokine-inducible SH2-containing protein (CISH) can be increased; however, SOCS1/3 and CISH overexpression directly leads to the inhibition of the downstream IL-7/STAT5 signalling pathway and the suppression of the proliferation and differentiation of naïve T cells while maintaining the activity of T cells [73]. Therefore, m6A RNA modification plays an important role in T-cell homeostasis. In addition, a new study showed that m6A preferentially deposited on the 5’ end of nascent transcripts under the action of heat shock proteins. In the UTR, e.g., in HSPH1, m6A modification was increased at the 5’-untranslated region (5’-UTR) to enhance cap-independent translation initiation [74]. In general, the association between 5’-UTR methylation and independent translation reveals the potential relationship between the heat shock response and m6A. We’ve sorted it out some m6A modification regulators and their major biological functions (Table 1).

**Table 1:** m6A modification regulators and their major biological functions

Category	m6A regulator	Main functions	References
Writers	METTL3	miRNA regulates mRNA methylation through sequence complementation and cell reprogramming	[47]
	METTL3	Mediates m6A modification involved in the regulation of spermatogenesis in mouse	[59]
	METTL3	Mediates m6A modification involved in the regulation of mouse cerebellar development	[63]
	METTL14, WTAP, VIRMA, RBM15, ZC3H13, METTL16	m6A methyltransferase complex component identification	[9]
	WTAP, METTL3, METTL14	WTAP, as a regulatory subunit, regulates the localization and substrate binding capacity of the catalytic subunits of the METTL3/METTL14 complex	[26]
Erasers	FTO	As the first discovered demethylase, FTO can catalyse the demethylation of m6Am and m1A	[75]
	FTO	Mediates m6A modification that can serve as a novel cis element to regulate mRNA splicing and adipocyte precursor cell differentiation	[48]
	ALKBH5	The second discovered demethylase; participates in the regulation of mRNA nuclear export and mouse sperm development	[33]
Readers	YTHDC1	YTHDC1 directly interacts with SRSF3 and SRSF10 to regulate alternative mRNA splicing	[35]
	YTHDC1	YTHDC1 interacts with SRSF3 and RNA nuclear export factor 1 (NXF1) to regulate mRNA nuclear export	[37]
	YTHDF1	YTHDF1 directly interacts with the translation initiation complex to promote the translation efficiency of m6A-modified RNA substrate	[34]
	YTHDF2	Mediates m6A modification involved in the regulation of mRNA decay	[44]
	YTHDF2, METTL3	Mediates m6A modification involved in the regulation of the differentiation of haematopoietic stem cells	[57]
	YTHDF1 and YTHDF3	YTHDF1 synergizes with YTHDF3 to regulate mRNA translation	[76]
	YTHDF2 and YTHDF3	YTHDF2 synergizes with YTHDF3 to mediate mRNA decay	[36]
	YTHDC2	YTHDC2 regulates mRNA translation or decay and mouse spermatogenesis	[38]
	IGF2BP1/2/3	Participates in m6A modification-mediated mRNA stability and translation	[42]

## Characteristics of m6A modification in gliomas

Gliomas are the most common primary malignant tumour in the central nervous system, with characteristics of high malignancy and poor prognosis. Their incidence accounts for 80% of all brain tumours. Despite the use of a variety of high-intensity treatment regimens, such as surgery combined with chemoradiotherapy, the median survival time of patients with GBM is still only 12–15 months, and only 3% -5% of patients have a survival time longer than 3 years [1–3]. Therefore, exploring the biological origin and occurrence of gliomas and finding potential diagnostic and therapeutic targets have been the focus of research in the field of molecular biology.

Li et al. recently reported that m^6^A methylation is reduced in glioma tissues, and that ectopically increasing m^6^A levels by METTL3 overexpression in one glioma cell line could impair its proliferation and migratory ability, while increasing apoptosis [77]. But they did not dig into the mechanism through which this epitranscriptomic modification may affect glioblastoma growth.

Cui’s group addressed the above point and described the involvement of m^6^A RNA methylation and of m^6^A-related proteins in glioblastoma in 2017 [78]. The model they chose were glioblastoma stem cells (GSCs), considered the initiating cells of glioblastoma, usually enriched in restricted niches and deemed responsible not only for glioblastoma onset but also for its resistance to therapy and eventual recurrence [79].

But on the other hand, Liu et al. found that WTAP expression predicts poor prognosis in malignant glioma patients [80]. As WTAP is a crucial interactor of the methyltransferase complex, so this works suggested that m^6^A modification related enzymes and m^6^A methylation processes may play an oncogenic role in glioma [81]. Visvanathan et al. published the first mechanistic work linking m^6^A modification and oncogenesis in glioblastoma. They studied the levels of m^6^A RNA methylation in three GSC lines and showed that they were reduced uponin vitro differentiation. Moreover, they also found that METTL3 mRNA was clearly more abundant in GSCs compared to counterparts [82, 83].

To sum up, the expression of m6A in glioma is different. This indicates that m6A modification may not only promote cancer but also inhibit it during the occurrence and development of glioma. So there’s been a lot of interests and researches from biomedical scientists.

### Writers and gliomas

GSCs are a group of cells with the ability to promote tumour growth and invasion and have strong resistance to both radiotherapy and chemotherapy. Therefore, the presence of GSCs indicates a poor prognosis for patients with GBM [84]. One study showed that in GSCs, the expression levels of METTL3 increased, and the expression levels of METTL14 and ALKBH5 decreased, while FTO did not show significant changes. By installing m6A on the SOX2 3’-untranslated region (3’-UTR), METTL3 mediates GSC maintenance and dedifferentiation by regulating the stability of SOX2 mRNA. The complete structure of METTL3 and human antigen R (HuR) is critical for maintaining this process. In addition, METTL3 knockdown inhibited GSC growth and neurosphere formation and reduced the expression levels of stem cell-specific markers, stage-specific embryonic antigen-1 (SSEA1), and glioma reprogramming factors (including POU class 3 homeobox 2 (POU3F2), oligodendrocyte transcription factor 2 (OLIG2), spalt like transcription factor 2 (SALL2) and SOX2). Of which, SOX2 has a high affinity for METTL3 [82].

The deep sequencing of m6A and mRNA showed that the knockdown of METTL3 and/or METTL14 led to the upregulation of oncogenes and genes coding downstream proteins, including ADAM metallopeptidase domain 19 (ADAM19), EPH receptor A3 (EPHA3), Kruppel-like factor 4 (KLF4) and tumour-inhibiting factors, resulting in the inhibition of GSC growth and self-renewal [85]. METTL3 overexpression or treatment with the FTO inhibitor MA2, the ethyl ester form of meclofenamic acid (MA), can cause an increase in m6A levels in GBM cells [78]. However, another study reported the opposite effect of METTL3 in GBM; this effect was related to a decrease in m6A levels during differentiation. Silencing METTL3 expression in GBM can significantly inhibit tumour growth and prolong mouse survival time, which is consistent with clinical observations that an increase in METTL3 expression is consistent with the poor survival of patients with GBM. Further studies on the mechanisms of action have shown that METTL3 is involved in the RNA processing and carcinogenic pathways of GSCs and has a variety of complexities. METTL3 plays a major role in m6A modification in GSCs and participates in the expression and alternative splicing of GSC-specific genes. In addition, METTL3 reduced A-to-I RNA editing by downregulating ADAR and ADAMRB1 and increased the editing abundance of C-U RNA by upregulating apolipoprotein B mRNA editing enzyme catalytic subunit 1 (APOBEC1) and APOBEC3A [59].

METTL3 expression is upregulated in GSCs and weakens during differentiation. SOX2 was identified as an important target of METTL3-mediated m6A, whereas METTL3 promoted the recruitment of HuR to m6A-modified SOX2 mRNA and enhanced SOX2 stability [85]. In addition, after the downregulation of METTL3 expression, GSCs showed strong radiosensitivity and a weak DNA repair capacity [82]. Therefore, the above studies also revealed that METTL3-mediated m6A modification was important in GSC maintenance and radiotherapy resistance. As a zinc finger protein, zinc finger CCCH-type containing 13 (ZC3H13), is also an important regulator in the m6A-METTL-associated complex (MACOM) and can anchor WTAP, virilizer and Hakai in the nucleus [78]. A recent study showed that the ZC3H13 mutation and retinoblastoma 1 (RB1) mutation could replicate human GBM in a mouse model. In addition, the ZC3H13 mutation also changed the gene expression profile of the RB1 mutant to enhance the resistance of GBM tumours to TMZ [86].

In addition, WTAP is overexpressed in GBM, and WTAP enhances the proliferation, migration, invasion, and tumourigenicity of GBM cells in xenografts by mediating the phosphorylation of epidermal growth factor receptor (EGFR) and protein kinase B (AKT). In addition, WTAP also regulates the expression of certain genes associated with cancer cell movement, such as chemokine ligand 2 (CCL2), CCL3, matrix metalloproteinase 3 (MMP3), lysyl oxidase like 1 (LOXL1), hyaluronic acid synthase 1 (HAS1) and thrombospondin 1 (THBS1) [81]. High WTAP expression is an independent prognostic factor that is positively correlated with age and World Health Organization (WHO) classification and indicates poor overall survival in GBM patients [80]. Cell-based experiments have shown that WTAP plays an important role in the miR-29a/Quaking isoform 6 (QKI-6) axis-mediated inhibition of cell proliferation, migration, and invasion as well as a downstream target for promoting GSC apoptosis [87].

In addition to the direct impact on pluripotent genes, MeRIP-seq analyses based on m6A-Seq techniques showed that m6A-modification peaks tend to be enriched in metabolic pathway-related transcripts [88]. METTL3 can cause changes through the downregulation of adenosine deaminase 1 (ADAR1) and apolipoprotein B mRNA expression, e.g., a reduction in editing events such as adenosine to inosine (A to I) and cytidine to uridine (C to U) (such as APOBEC3A) in GSCs [89]. In addition, gene ontology analysis indicated that the direct target of METTL3 seems to be enriched in some major oncogenic pathways, including the Notch signalling pathway, vascular endothelial growth factor (VEGF) signalling pathway, angiogenesis, glycolysis and the Hedgehog signalling pathway; the indirect target is enriched in the RAS pathway, mitogen-activated protein kinase (MAPK) pathway, G-protein coupled receptor (GPCR) pathway, cadherin signalling pathway and cell cycle [89]. In addition, in GSCs, METTL3-mediated m6A modification can also affect expression levels of serine and arginine rich splicing factors (SRSF) by upregulating BCL-X or nuclear receptor corepressor 2 and can prevent YTHDC1-dependent nonsense-mediated mRNA decay (NMD) [88]. Compared with protein-coding genes, METTL3-mediated m6A-tagged lncRNAs are also highly expressed in GSCs. Furthermore, the m6A marker in the 3’-UTR appears to block the binding process of microRNA-related genes in GSCs [89].

In summary, m6A writers are critical for the occurrence and development of GBM, and most are upregulated in GBM and show carcinogenic effects by regulating specific signalling pathways, especially helping to maintain cell stemness. However, some opposite findings indicate that the expression of some writers in GBM is downregulated and that some writers may have anticancer properties. Therefore, the existence of this conflict provides more research possibilities on the role of m6A modification-related methylation in the biological pathogenesis of gliomas.

### Erasers and gliomas

Similar to writers, m6A erasers also play vital roles in GBM. The latest research shows that ALKBH5 is elevated in GSCs, enhancing cell self-renewal, proliferation and tumourigenicity [78]. In terms of a mechanism, ALKBH5 demethylates m6A-modified bases and enhances the expression level of the key target gene forkhead box protein M1 (FOXM1) in GBM patients by reducing the abundance of m6A in the target mRNA transcript (especially in the 3’-UTR) [90].

As an important functional target of ALKBH5, FOXM1 overexpression can reverse the function of ALKBH5 or inhibit FOXM1 long noncoding RNA antisense (FOXM1-AS) and restore GSC tumour growth. FOXM1-AS is a lncRNA on human chromosome 12 that is opposite to and partially overlaps with FOXM1. FOXM1-AS can promote the interaction between ALKBH5 and FOXM1 nascent transcripts, thereby promoting the recruitment of HuR. In general, under the combined action of FOXM1-AS, ALKBH5 enhances the self-renewal and proliferation of GSCs by regulating the expression of FOXM1 and promoting the occurrence and development of GBM [90]. On the other hand, ALKBH5 knockdown inhibits the proliferation of GSCs, while wild-type ALKBH5 rescues the proliferation of GSCs. After ALKBH5 knockdown, the m6A level in nascent FOXM1 transcripts is elevated, and the binding of FOXM1 pre-mRNA to HuR is reduced; therefore, the recruitment of HuR to m6A-modified RNA is crucial for stabilizing FOXM1 mRNA [90].

Su’s study showed that the inhibition of FTO hindered the self-renewal ability and carcinogenicity of GBM stem cells *in vitro* and in mouse models. FTO plays carcinogenic roles through maintaining the stability of gliomas, especially the stability of oncogene homologues (c-Myc) and CCAAT-enhancer-binding protein-α (CEBPA) transcripts in IDH1/2 mutant gliomas. In addition, the inhibitory effect of MA2 on FTO significantly increases the tumourigenicity of GSC-transplanted mice [75, 78]. The above evidence also potentially reveals that FTO may be a promising target for the drug treatment of GBM.

### Readers and gliomas

#### YTHDF and YTHDC family

The YTHDF and YTHDC series are the most important code readers for m6A modification, as they include a YTH domain that can bind to RNA. They exhibit different functions: YTHDC1 mediates mRNA splicing; YTHDF2, YTHDF3 and YTHDC2 mediate mRNA decay; YTHDF1, YTHDF3 and YTHDC2 mediate mRNA translation; and YTHDC2 and YTHDC2 mediate RNA structure [91]. Recently, a study by Li et al. showed that these important m6A-related proteins are involved in the development of GBM. After YTHDC1 knockdown, proliferation was significantly reduced in U87 cells with METTL3 overexpression but not in control cells. In addition, for the W377A/W428A mutant with METTL3 overexpression, YTHDC1 failed to promote the ability of U87 cells to form spheres, indicating that YTHDC1 relies on its m6A binding activity to promote the functional phenotype of GBM [88].

#### IGF2BP family

Another m6A reader protein family, the IGF2BP family (IGF2BP1/2/3), inhibits the decay of m6A-modified transcripts and promotes their translation [42]. For a long time, these proteins have been considered important regulatory factors in the pathogenesis of GBM, even though some functions are not directly related to carcinogenesis. For example, IGF2BP1 can promote the proliferation and invasion of GBM cells by stabilizing the mRNA transcripts of its target genes, including c-Myc, Ki-67, phosphatases and tensin homologue (PTEN) and CD44 (cell-surface glycoprotein 44) [92, 93].

Studies have shown that IGF2BP2 is upregulated in GBM tissues, promoting the proliferation, migration, invasion, and epithelial-mesenchymal transition (EMT) of GBM cells through the regulation of insulin-like growth factor 2 (IGF2) activity while further activating the phosphoinositide 3-kinases (PI3K)/AKT signalling pathway. In addition, IGF2BP2 inhibition can cause an increase in the sensitivity of GBM to TMZ [94]. Another study showed that IGF2BP2 binds to the miRNA recognition elements (MREs) of lethal-7 (let-7) and blocks let-7 target gene silencing, which is LIN28-independent, including high mobility group AT-hook 1 (HMGA1), HMGA2, cyclin D2 (CCND2) and ribonucleotide reductase regulatory subunit M2 (RRM2), thus maintaining the stemness of GSCs [95]. During this process, IGF2BP2 is responsible for miRNA maturation, and it also interacts with lncRNAs such as hypoxia inducible factor 1 alpha-antisense RNA 2 (HIF1A-AS2), which can specifically induce hypoxia to maintain the expression level of its target gene HMGA1, eventually promoting GSC proliferation, self-renewal and the reprogramming of hypoxia-dependent molecules [96]. In addition, IGF2BP2 also interacts with mRNAs and proteins. For example, IGF2BP2 can bind to several mRNAs, including mitochondrial cytochrome C oxidase subunit 7B (COX7B), NADH dehydrogenase iron-sulfur protein 7 (NDUS7), and NADH dehydrogenase, to promote oxidative phosphorylation in GSCs [97].

The mRNA and protein expression levels of IGF2BP3 are upregulated in GBM but not significantly upregulated in low-grade astrocytomas [98]. According to the gene expression microarray analysis of 9 pilomyxoid astrocytoma (PMA) and 13 pilocytic astrocytoma (PA) from lower and upper loci, the expression level of IGF2BP3 in malignant astrocytoma is significantly increased [99]. In addition, gene chip analysis in glioma cells indicated that IGF2BP3 mediates the association between direct targets at the transcriptome level and processes related to the cell cycle as well as the association between direct targets at the translatome level and apoptosis-related pathways [100]. IGF2BP3 also induced EMT by downregulating the expression of E-cadherin and upregulating the expression levels of N-cadherin, vimentin and MMP-9, thereby promoting cell proliferation, migration and invasion [101]. In addition, IGF2BP3 activates the PI3K/MAPK pathway by binding to the 5’-UTR of IGF-2 mRNA to activate its translation, thereby promoting cell proliferation, anchorage-independent growth, invasion, and chemoresistance [98]. IGF2BP3 also stimulates the migration of glioma cells by enhancing the translation of p65 (RELA), which is a subunit of the nuclear factor-kappa B (NF-κB) heterodimer, and p65 can also transcriptionally activate IGF2BP3 to form a feedback loop [102].

#### hnRNPA2B1 and hnRNPC family

A study of SOX2 protein interactions showed that hnRNPA2B1 and hnRNPC could interact with SOX2 in GBM, suggesting that they might play a key role in maintaining the stemness of GSCs [103]. Heterogeneous nuclear ribonucleoproteins A2/B1 (hnRNPA2B1) is an important regulator of mRNA metabolism and transport in cells. Its downstream protein is highly expressed in glioma tissues and is associated with the histologic glioma grade and a poor prognosis [104]. hnRNPA2B1 may promote the proliferation, migration, and invasion of GBM cells through the downregulation of tumour suppressor factors, enhance chemoresistance to TMZ, and protect cells from apoptosis and damage caused by reactive oxygen species (ROS) [105].

**Fig. 3:**
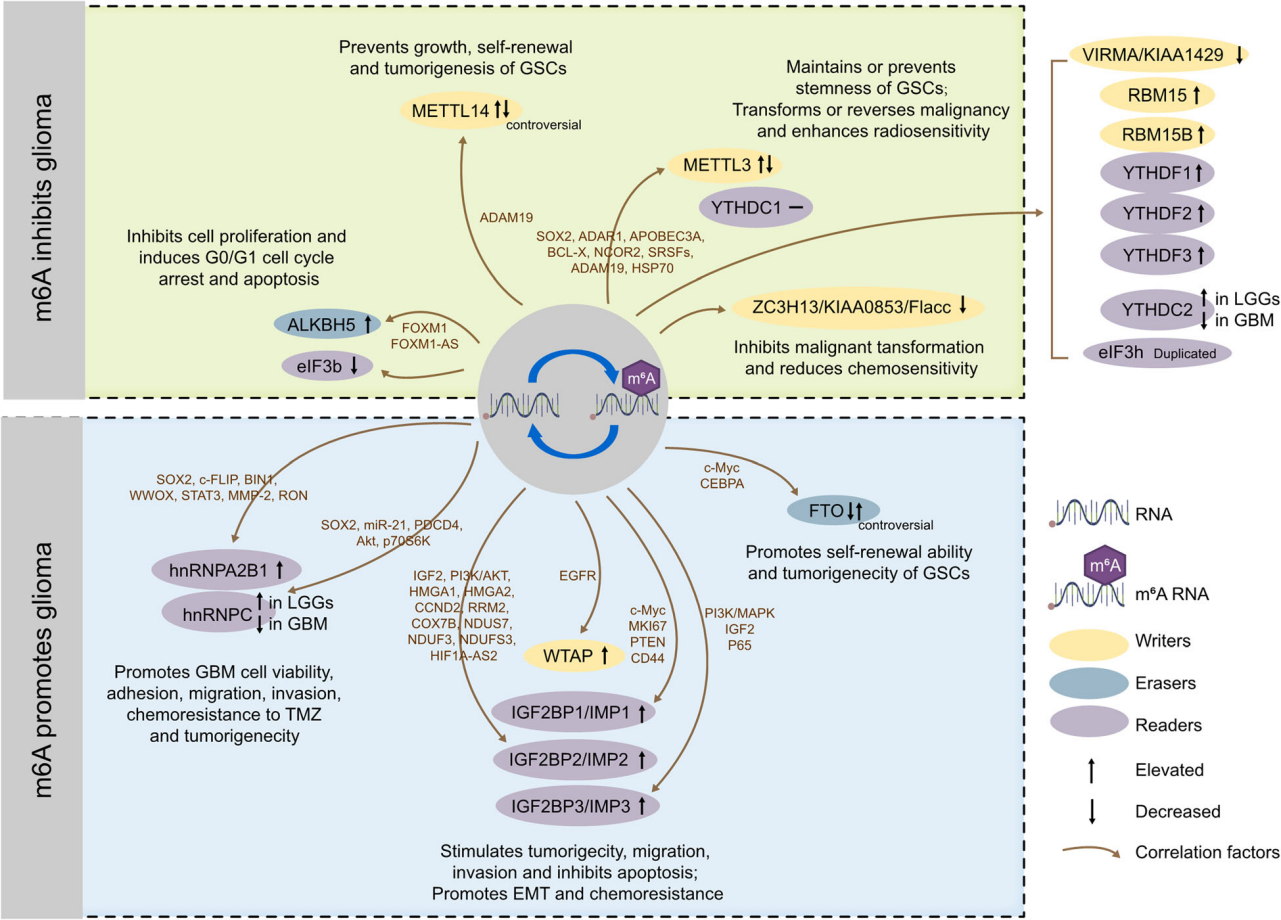
The potential roles of RNA m6A modification in glioma progression. They are reflected in the regulation of tumor-associated factors. m6A promotes glioma progression by enhancing oncogene expression and inhibiting tumor suppressor gene expression. m6A hampers glioma progression by inhibiting oncogene expression and enhancing tumor suppressor gene expression

In addition, hnRNPC is an important physiological modulator for 3’-UTR processing and miRNA maturation as well as a modulator for neoplastic disease [106]. hnRNPC has higher expression levels in higher-grade GBM; hnRNPC directly binds to miR-21 (mainly pri-miR-21) and promotes miR-21 processing against programmed cell death 4 (PDCD4), which is an important regulator of cell apoptosis and survival. PDCD4 subsequently promotes the activation of AKT and p70 S6 kinase (p70S6K) and then enhances the migration and invasion activity of tumour cells, increases cell proliferation, and protects GBM cells from apoptosis [107]. These results indicate that hnRNPA2B1 and hnRNPC may be important m6A “readers” that are closely related to the biogenesis of GBM. Finally, we mapped the mechanism of action of m6A-modified important proteins associated with the biological behavior of glioma cells (Fig. 3).

## Clinical significance of m6A modification in gliomas

### m6A modification and disease diagnosis

Epigenetic alterations are considered promising markers for GBM diagnosis. In addition, some epigenetic statuses indeed explain the outcome of GBM [108–111]. For example, the epigenetic silencing of O-6-methylguanine-DNA methyltransferase (MGMT) significantly affected the TMZ treatment effect in GBM patients. Therefore, the methylation status of the MGMT promoter is an important biological marker used to predict GBM patient survival and GBM response to TMZ [112, 113].

As an important chemical modification to RNA, regular changes in m6A can also predict the prognosis of patients with GBM or be used for the diagnosis of GBM. Related studies have shown that m6A in RNA from peripheral blood is a biomarker of gastric cancer [114]. The methylation level of miRNAs is also a potential diagnostic biomarker for early gastrointestinal cancer [115]. ALKBH5 and FTO, which act as m6A erasers, have also been proven to be prognostic biomarkers for patients with renal clear cell carcinoma [116]. Therefore, for central nervous system tumours, the identification of modifications to RNA from peripheral blood or cerebrospinal fluid may be a promising method for GBM diagnosis.

### Expression of the m6A modifier is related to the clinicopathological features of gliomas

Considering the important biological functions of m6A modification-related proteins involved in the occurrence and development of tumours, some researchers conducted genomic profiling-based data mining and bioinformatics analysis to systematically investigate the relationship between each type of m6A modulator and pathological features of gliomas, including WHO classification, isocitrate dehydrogenase (IDH) classification and 1p/19q status. The results showed that the expression level of most m6A modulators was significantly correlated with the WHO histologic grade and corresponding classification. Through quantitative analysis, the expression levels of WTAP, YTHDF, ALKBH5 and FTO were significantly correlated with the histologic grade, the expression abundances of WTAP, YTHDF and ALKBH5 were positively correlated with the histologic grade, and the expression abundance of FTO was negatively correlated with the histologic grade [117].

### Potential therapeutic significance of m6A modification in gliomas

The covalent modification of DNA, histones and other proteins has shown potential as a cancer treatment. Some epigenetic drugs, such as suberanilohydroxamic acid (SAHA), romidepsin, Belinostat and Panobinostat, have been approved by the U.S. Food and Drug Administration (FDA). Moreover, chidamide has also been approved by the Chinese National Medical Products Administration for the treatment of certain T-cell lymphomas or multiple myeloma. In addition, many other clinical trials of epigenetic drugs are also underway [118]. Drugs targeting protein posttranslational modifiers (such as E3 ubiquitin-protein ligase), including S-phase kinase associated protein 2 (SKP2), speckle type BTB/POZ protein (SPOP), cellular inhibitor of apoptosis 2 (cIAP) and anaphase-promoting complex/cyclosome (APC/C), have also been evaluated in clinical practice and preclinical applications [119].

m6A is the most common covalent modification of RNA at the posttranscriptional level. Scientists are exploring its therapeutic potential in malignant tumours such as GBM. Currently, some pharmaceutical companies are planning to develop a METTL3-METTL14 complex and a small molecule inhibitors of ADAR [120]. Several compounds with piperidine or piperazine rings have very high cooperative binding efficiency with METTL3-METTL14-WTAP complexes, thereby activating RNA methylation [121]. Recently, METTL3 small molecule inhibitors have also shown the ability to inhibit the progression of acute myeloid leukaemia (AML) in the body [122]. Based on the abovementioned latest research, it is believed that m6A modification-related targeted therapies for gliomas will be developed.

### m6A modification and prognosis of patients with gliomas

Some researchers conducted a univariate Cox regression analysis on the expression levels of m6A-related proteins in the Chinese Glioma Genome Atlas (CGGA) database and found that high-risk genes mainly include ALKBH5, YTHDF1, YTHDF2, HNRNPC, RNA binding motif protein 15 (RBM15), KIAA1429 and WTAP and that the protective genes are mainly FTO, YTHDC1, ZC3H13 and METTL3. Among the genes with predictive value in adult low-grade gliomas (LGGs), the expression levels of YTHDF2, WTAP, ALKBH5, RBM15, KIAA1429, HNRNPC, YTHDF1 and FTO were significantly correlated with the overall survival (OS) of IDH-mutated glioma patients. In summary, YTHDF2, KIAA1429, HNRNPC, and YTHDF1 had predictive value in LGB with wild-type IDH, and FTO, YTHDF2, and RBM15 had predictive value in GBM with wild-type IDH; FTO also had predictive value in GBM with mutated IDH [117].

In addition, another univariate and multivariate Cox regression analysis showed that risk score, 1p/19q sequence, IDH status, age and WHO classification were all associated with OS. After including these factors in the multivariate Cox regression, risk scores and WHO classification were still significantly correlated with OS (P < 0.001). Further multiple regression analysis showed that risk score (P = 0.027), IDH status (P < 0.001), age (P < 0.001) and WHO classification (P = 0.005) were still significantly correlated with OS. In summary, the risk score derived from the RNA m6A modulator can independently predict the prognosis of glioma patients. In addition, for WHO grades II and III gliomas, the OS of patients with high risk scores was significantly shorter than that of patients with lower risk scores. Through comprehensive analysis of the WHO classification, risk score also had predictive value for gliomas. In addition, according to the CGGA and The Cancer Genome Atlas (TCGA) databases, GBM patients with high risk scores were also more sensitive to TMZ treatment and had relatively lower drug resistance [117].

## Discussion and outlook

There is a correlation between the degree of m6A RNA methylation and the differentiation of GSCs and between the effect of METTL3 knockdown on in *vitro* self-renewal and *in vivo* tumourigenicity of glioblastoma in mice. GSCs and their derived tumours can be divided into subtypes based on different characteristics and different aggressiveness and responsiveness to therapy. Research on m6A modification and oncology is still an emerging field. Therefore, all cited papers are based on a very limited number of cell models, and the results may be affected by variability. One study analysed 7 GSC lines using RNA-seq and compared the normal human brain with an established GBM cell line; the study did not find any clear evidence (e.g., author, reader, eraser, etc.) to prove the differential expression of mRNA coding proteins involved in m6A methylation. However, the controversial results were not discredited, and some genes or proteins involved in mRNA expression were still considered potential prognostic markers of GBM.

However, for this mechanism, it seems more necessary to clarify which mRNAs are hypermethylated or hypomethylated and which readers identify methylation under certain circumstances. It has been demonstrated that IGF2BPs are carcinogenic in glioblastoma, especially in GSCs, in which their function as specific target mRNA stability enhancers is related to their ability to induce or maintain glioblastoma growth. By correlating these data with currently known m6A readers, we can gain a deeper understanding of other molecular mechanisms by which these proteins may be involved in glioblastoma tumourigenesis.

Wu et al. recently published a study demonstrating that the m6A reader PRRC2a plays a key role in the formation of oligodendrocytes and myelination by binding to methylated adenosine in glioblastoma cells. This binding can stabilize OLIG2 mRNA, thereby stabilizing Olig2 protein production. In contrast, FTO demethylates OLIG2 mRNA at this site, thereby promoting its decay and Olig2 protein consumption. This study focused on oligodendrocytes in physiological and pathologically low-myelination conditions, but it should be noted that in addition to playing a major role in the development of oligodendrocytes, Olig2 protein also plays a role in GBM cell reprogramming and the genotoxicity and phenotypic plasticity of tumours. In addition, Olig2 expression is a recognized marker for the proneural subtype of GBM. More importantly, Olig2 protein is synergistic with Sox2, Pou3f2 and Sall2 and is a key transcription factor of glioma initiating cells [123]. This observation is only an example that partially elucidates how the proteins involved in transcriptome regulation are transferred under specific pathological conditions, especially in GBM. This field is worthy of further in-depth studies. Based on the information regarding transcriptome modifications in gliomas obtained from recent studies, we may have only revealed the tip of the iceberg, and in the future, we will definitely find more complex mechanisms of precise regulation of the transcriptional fate of glioma cells.

## Conclusions

In summary, although the correlation between m6A modification and oncology, as a hotspot in the field of biomedicine, has been extensively explored, most studies concentrated on gene sequencing analysis, differential expression analysis, and modification site analysis. There are few studies on the functional phenotypes and mechanisms of action at the cell level, but studies in this field are likely to be key to revealing the origin of tumours, especially the origin and development of malignant tumours. With the rapid development of molecular biology technology, especially single-cell sequencing and third-generation sequencing, the answer of how RNA methylation modification affects the biological behaviour of gliomas will gradually become clear. It provides new insights for the early diagnosis of, histologic grading of, and targeted therapy for gliomas. It also points to a new direction for the study of other neoplastic diseases. As a star molecule in RNA post-transcription modification research, exploring m6A modification might decipher the molecular mechanism of many diseases, serving as a new medical breakthrough anticipated by biomedical scientists.

## Data Availability

The datasets used and analysed during the present study are available from the corresponding author on reasonable request.

## Abbreviations

GBM: Glioblastoma
TMZ: Temozolomide
m6A: N6-methyladenine
mRNA: Messenger RNA
m6A-seq: M6A RNA immunoprecipitation sequencing
HPLC-MS/MS: High-performance liquid chromatography-tandem mass spectrometry
MeRIP-seq: Methylated RNA immunoprecipitation followed by high-throughput sequencing
PA-m6A-Seq: Photo-crosslinking-assisted m6A-sequencing
SCARLET: Site-specific cleavage and radioactive-labelling followed by ligation-assisted extraction and thin-layer chromatography
miCLIP: M6A individual nucleotide resolution crosslinking immunoprecipitation
miRNA: Micro RNA
lncRNA: Long noncoding RNA
circRNA: Circular RNA
rRNA: Ribosomal RNA
tRNA: Transfer RNA
snoRNA: Small nucleolar RNA
METTL3: Methyltransferase like 3
WTAP: Wilms' tumour 1-associating protein
SAM: S-adenosylmethionine
FTO: Fat mass and obesity-associated gene
ALKBH5: AlkB homologue 5
YTHDF1: YTH N6-methyladenosine binding protein 1
YTHDC1: YTH domain containing 1
SRSF3: Serine-rich splicing factor 3
IGF2BP: Insulin-like growth factor-II mRNA binding protein
P body: Processing body
EHT: Endothelial-to-haematopoietic transition
Notch1: Notch homologue protein 1
HSPC: Haematopoietic stem cell
ESC: Embryonic stem cell
ZFP217: Zinc finger protein 217
SOX2: Sex determining region Y box 2
KLF4: Kruppel-like factor 4
HE: Haematoxylin-eosin
IL-7: Interleukin-7
STAT5: Signal transducer and activator of transcription 5
CGC: Cerebellar granule cell
EGL: External granular layer
SOCS: Suppressor of cytokine signalling
CISH: Cytokine-inducible SH2-containing protein
5ʹ-UTR: 5ʹ-untranslated region
GSC: Glioblastoma stem cell
3ʹ-UTR: 3ʹ-untranslated region
HuR: Human antigen R
SSEA1: Stage-specific embryonic antigen 1
POU3F2: POU class 3 homeobox 2
OLIG2: Oligodendrocyte transcription factor 2
SALL2: Spalt like transcription factor 2
ADAM19: ADAM metallopeptidase domain 19
EPHA3: EPH receptor A3
MA: Meclofenamic acid
APOBEC1: Apolipoprotein B mRNA editing enzyme catalytic subunit 1
ZC3H13: Zinc finger CCCH-type containing 13
MACOM: M6A-METTL-associated complex
RB1: Retinoblastoma 1
EGFR: Epidermal growth factor receptor
CCL2: Chemokine ligand 2
MMP3: Matrix metalloproteinase 3
LOXL1: Lysyl oxidase like 1
HAS1: Hyaluronic acid synthase 1
THBS1: Thrombospondin 1
WHO: World Health Organization
QKI-6: Quaking isoform-6
ADAR1: Adenosine deaminase 1
VEGF: Vascular endothelial growth factor
MAPK: Mitogen-activated protein kinase
GPCR: G-protein coupled receptor
SRSF: Serine and arginine rich splicing factor
NMD: Nonsense-mediated mRNA decay
FOXM1: Forkhead box protein M 1
CEBPA: CCAAT-enhancer-binding protein-α
PETN: Phosphatases and tensin homologue
CD44: Cell-surface glycoprotein 44
EMT: Epithelial-mesenchymal transition
IGF2: Insulin-like growth factor 2
PI3K: Phosphoinositide 3-kinases
MRE: MiRNA recognition element
let-7: Lethal-7
HMGA1: High mobility group AT-hook 1
CCND2: Cyclin D 2
RRM2: Ribonucleotide reductase regulatory subunit M 2
HIF1A-AS2: Factor 1 alpha-antisense RNA 2
COX7B: Cytochrome C oxidase subunit 7 B
NDUS7: NADH dehydrogenase iron-sulfur protein 7
PMA: Pilomyxoid astrocytoma
PA: Pilocytic astrocytoma
NF-κB: Nuclear factor-kappa B
hnRNPA2B1: Heterogeneous nuclear ribonucleoproteins A2/B1
ROS: Reactive oxygen species
PDCD4: Programmed cell death 4
p70S6K: P70 S6 kinase
MGMT: Methylguanine-DNA methyltransferase
IDH: Isocitrate dehydrogenase
SAHA: Suberanilohydroxamic acid
FDA: Food and drug administration
SKP2: S-phase kinase associated protein 2
SPOP: Speckle type BTB/POZ protein
cIAP: Cellular inhibitor of apoptosis
APC/C: Anaphase-promoting complex/cyclosome
AML: Acute myeloid leukaemia
CGGA: Chinese Glioma Genome Atlas
RBM15: RNA binding motif protein 15
LGG: Adult low-grade glioma
OS: Overall survival
TCGA: The Cancer Genome Atlas
